# Surface phonons in topological insulator Bi_2_Te_3_ investigated by Brillouin light scattering

**DOI:** 10.1038/s41598-020-68690-z

**Published:** 2020-07-16

**Authors:** Aleksandra Trzaskowska, Boguslaw Mroz

**Affiliations:** Faculty of Physics, Adam Mickiewicz University, Uniwersytetu Poznańskiego 2, 61-614 Poznan, Poland

**Keywords:** Surfaces, interfaces and thin films, Mechanical properties, Topological insulators

## Abstract

High resolution Brillouin spectroscopy was used for the first time to study the dispersion and anisotropy of surface phonons in the single crystal of topological insulator Bi_2_Te_3_. Two surface acoustic waves have been observed, which distinguishes this material from other metals or nontransparent materials. The modes were assigned as Rayleigh waves. The obtained results were then simulated by Finite Element Method. The layered structure of the unit cell proposed in simulation reproduced quite well experimental results of the modes dispersion and anisotropy.

## Introduction

Topological insulators (TI) have exotic conductive states on their surface whereas their interior remains insulating^1^. These surface states, which arise from a strong spin–orbit coupling, are not sensitive for impurities or lattice defects. As a results the lossless electronic transport is observed which may be used in new spintronic devices^2–4^.

The development of angle resolved photoemission spectroscopy (ARPES) allowed for the direct observation of the electronic dispersion of different materials, including TI, as well as the strength of electron phonon coupling (EPC)^5,6^. The EPC produces the abrupt change in the screening of lattice vibrations by conduction electron, leading to the well-known Kohn anomaly^7^ manifested by a discontinuity in the derivative of the surface phonons dispersion relation that occurs at certain high symmetry points of the first Brillouin zone.

Giraud and Egger analyzed the deformation potential linking Dirac fermions and acoustic phonons which allowed them to determine dispersion curves for low-frequency acoustic modes and their contribution to the electrical resistivity^8,9^.

Another model, which inspired us to run the presented experiment was proposed by Thalmeier^10^. He replaced the global normal to the surface in the effective Dirac Hamiltonian by the local normal which depends on the position on the surface. This allowed to couple the Dirac states to the surface phonons dynamic.

The family of stoichiometric 3D topological insulatorsBi_2_Te_3_, Bi_2_Se_3_ and Sb_2_Te_3_ is known to possess the surface states consist of a single Dirac cone at the Brillouin zone center. In Bi_2_Se_3_ the deep Kohn anomaly was revealed in the helium-atom-scattering experiment (HAS)^11^.

In our recent paper^12^ it was shown that the electron–phonon interaction at a conducting interface between a topological insulator thin film and a semiconductor substrate can be directly probed by means of high-resolution Brillouin light scattering (BLS).

Apart from classical crystallographic structure Bi_2_Te_3_ shows a superlattice built of quintalayers separated by “empty” van der Waals regions. The superlattice may be treated as a kind of nanocomposite creating the bulk material. Thus, one could expect some interesting features in elastic material response. Since BLS penetrate several dozen of lattice constants the natural consequence was to investigate Bi_2_Te_3_ single crystal surface elastic properties in the GHz frequency range. The additional motivation was lack of experimental data covering this frequency range. So far elastic properties of Bi_2_Te_3_ have been studied using the ultrasonic method^13,14^ (MHz range) and local static nanoindetation method^15^. Herein, we report experimental studies of surface acoustic waves of Bi_2_Te_3_ bulk single crystals. The high resolution Brillouin scattering was used, for the first time, to determine the dispersion and anisotropy of surface acoustic waves (SAW). The results of experiment are supported by the simulations of surface phonons frequencies and their anisotropy with the use of finite element method (FEM).

## Results

**Figure 1:**
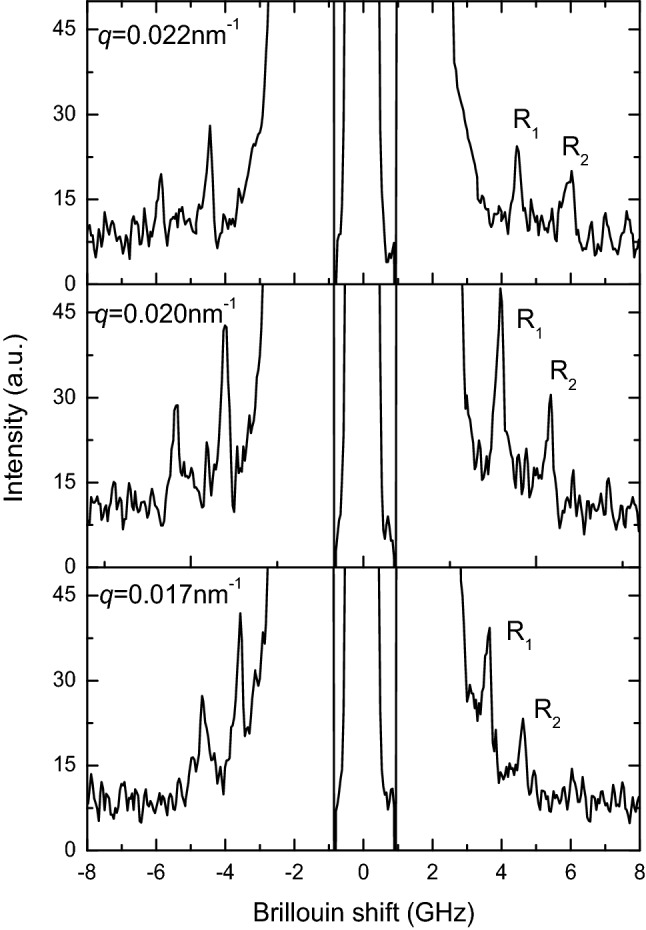
Brillouin spectra of surface acoustic waves (SAW) obtained from Bi_2_Te_3_ (0001) plane with two modes (denotes as R_1_ and R_2_) for different wave vectors.

The examples of spectra obtained for different wave vectors are shown in Fig. 1.

The most recognized surface excitations are: Rayleigh, Lamb, Love, Sezawa, and Stoneley waves^16^. The occurrence of the last four types of surface waves is possible when a layer of another material is applied to the surface of a given medium. We are then dealing with a layer—substrate system. The mutual ratio of the transverse wave velocity in the layer and in the substrate as well as the ratio of the thickness of the applied layer and the specific components of the tensor of elastic properties determines the type of wave observed^17–20^. This of course is not our case since our sample is a bulk single crystal.

**Figure 2:**
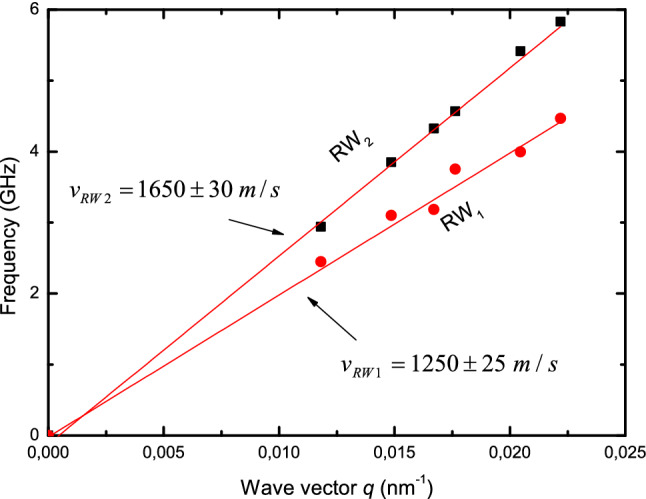
The linear dispersion relation for RWs obtained from Bi_2_Te_3_ (0001) plane. The measurements were done along [$0\overline{1}10]$ direction.

What is new in above presented results is the simultaneous appearance of two modes R_1_ and R_2_ in the collected spectra. Taking into account the linear angular dependence of frequencies of R_1_ and R_2_ modes one it may be concluded that we are dealing with Rayleigh waves (RW_1_ and RW_2_) propagating with velocities 1,250 and 1,650 m/s respectively (Fig. 2).

The slower one (RW_1_) is pure surface wave whereas the RW_2_ mode exhibits features of so called pseudo surface acoustic wave but only on small part of velocity diagram (we discuss it more precisely in Supporting Informations).

**Figure 3:**
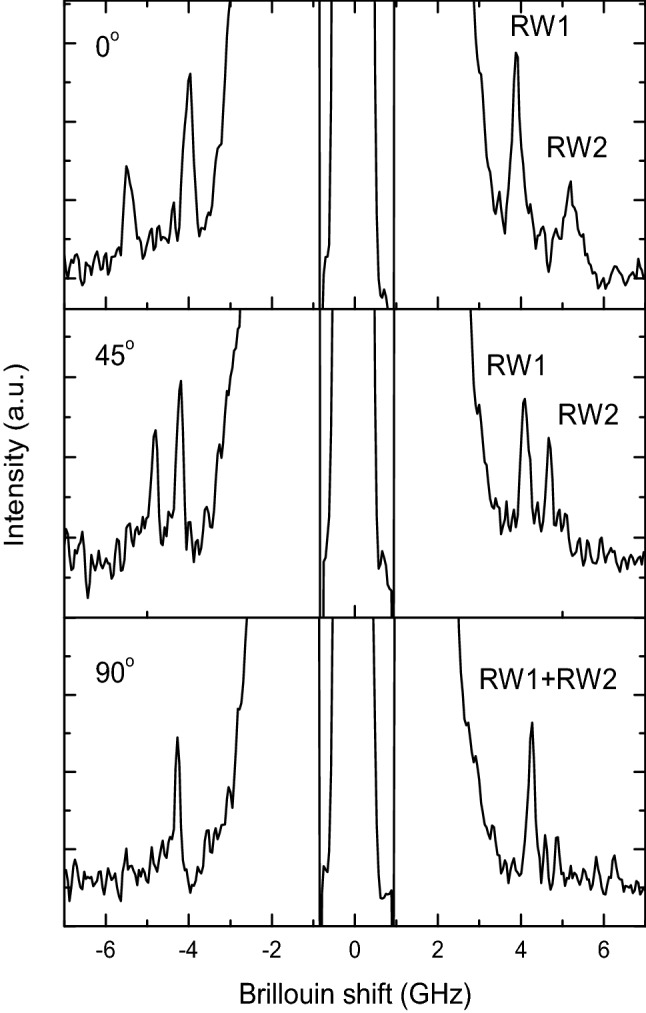
The evolution of Brillouin spectra in course of anisotropy measurements. The Θ angle is fixed at 60°.

The angular dispersion of the two observed Rayleigh waves were also determined. The evolution of frequencies of RW_1_ and RW_2_ are presented in the Fig. 3. The spectra were collected for fixed Θ = 60° which correspond to the wave vector *q* = 0.019 nm^−1^.

**Figure 4:**
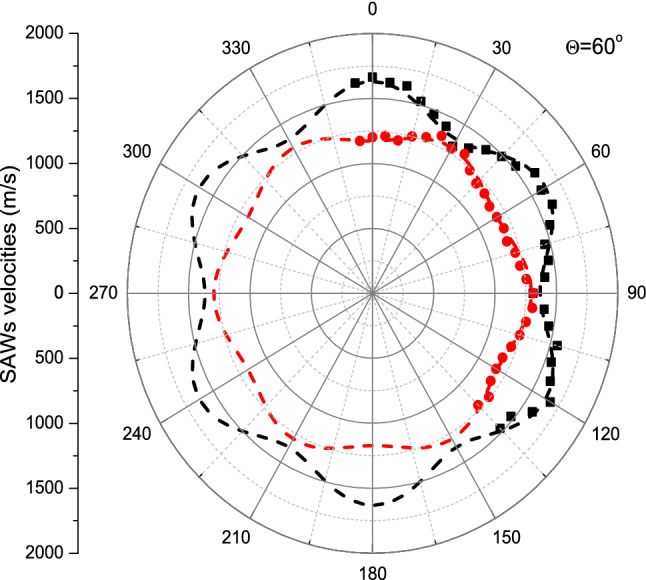
Anisotropy of two Rayleigh waves (RW_1_-red circles and RW_2_- black squares) on (0001) plane of Bi_2_Te_3_. The dashed lines are guided on eye.

It is evident that anisotropy of the both modes reflect the trigonal symmetry of Bi_2_Te_3_. This can be clearly seen in the velocity diagram (Fig. 4) constructed on the basis of obtained results.

The frequency of the modes studied varies—the angular dispersion of the modes frequency were anisotropic for both RW_1_ and RW_2_ (Fig. 4). The angular dispersions of RWs are rotated of 30 degrees relative to each other.

## Discussion

The Bi_2_Te_3_ exhibit a layered rhombohedral lattice structure (space group $\overline{3}m$) with three quintuple (QL)—[Te(I)-Bi-Te(II)-Bi-Te(I)]—stacks forming a unit cell. Each quintuple layer consists of five atoms with two equivalent Te atoms, two equivalent Bi atoms and a third Te atom^5^. The coupling is strong between two atomic layers within one QL while two neighboring QLs the coupling is much weaker mainly because of the van der Waals (VdW) bonding^21,22^. Because of the layered structure, the crystals show cleavage plane perpendicular to the [0001] direction.

The FEM method is based on division of the medium into small elements for which is possible to approximate the solution of wave equation by a linear function, which permits a transformation of the differential equation into a set of algebraic equations^23^.

**Figure 5:**
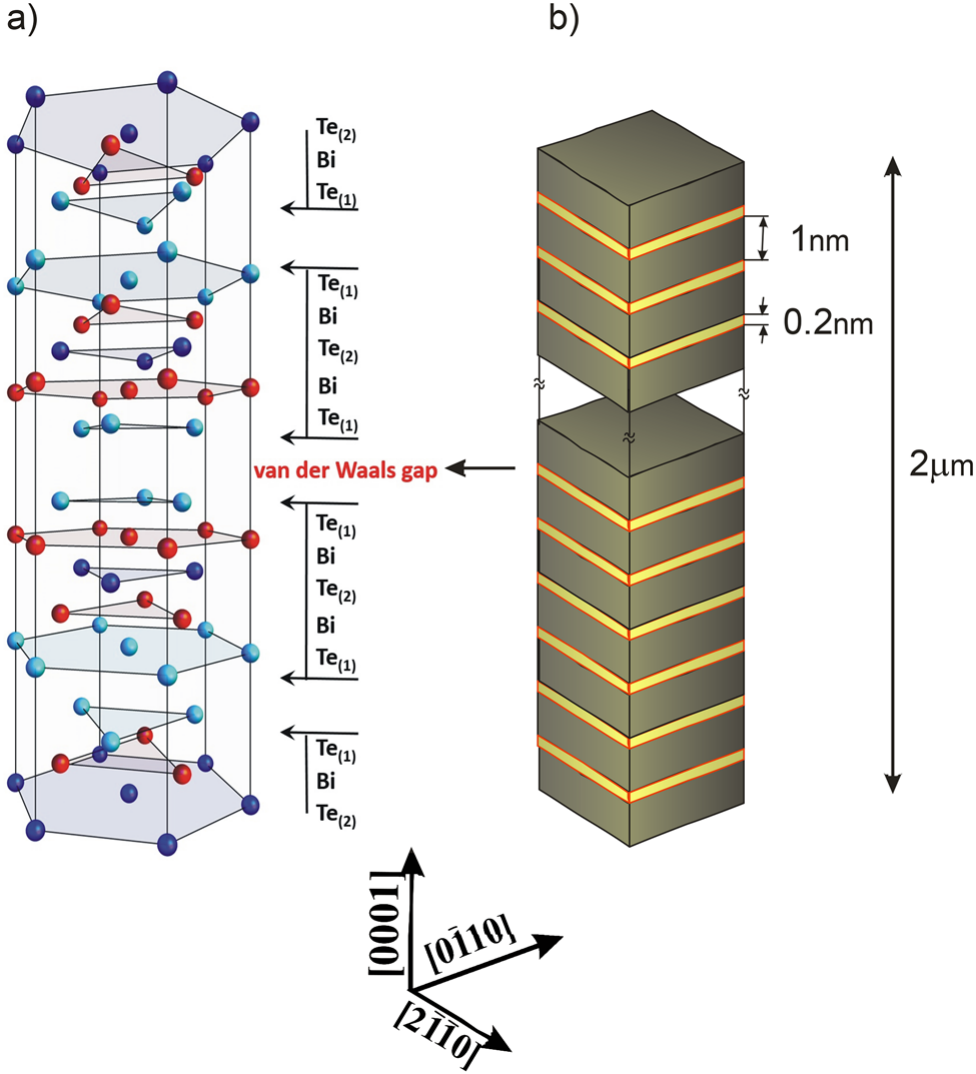
The unit cell of Bi_2_Te_3_ (**a**) and FEM unit cell used in simulations (**b**).

According to the results of x-ray investigations the unit cell used for FEM simulations been constructed as follows: the section of solid layer (brown) is separated with “empty” yellow van der Waals regions (see Fig. 5). FEM requires an accurate determination of elastic properties of each component so the VdW regions were thus replaced by an air layer to feel in the empty space (or sparse matter). The thickness of QL was set to be 1 nm whereas VdW 0.2 nm^24^.

The boundary conditions of the bottom wall were fixed and the height of the elementary cell used in the simulations was correlated with the wavelength of light used in experiment just to avoid the unwanted reflection of simulated waves from the bottom of the sample. More information can be found in the Supporting Information.

For such structure FEM simulations gave the set of two Rayleigh waves propagating in the direction [$0\overline{1}10]$ on the plane (0001) with velocities 1,220 and 1,610 m/s (see Table 1).

**Table 1:** The values of the SAW velocities propagated in [$0\overline{1}10]$ direction on the (0001) plane of bismuth telluride obtained from FEM simulation and Brillouin scattering experiment.

	υ_RW1_(m/s)	υ_RW2_ (m/s)
FEM simulations	1,220	1,610
Presented experiment	1,250 ± 25	1,650 ± 30

Within the accuracy of measurements the velocities of these waves are the same as those detected in BLS experiment.

FEM allowed additionally for calculations of surface acoustic wave velocity from the known set of elastic constants. It is obvious that the data in Table 3 refer to the Bi_2_Te_3_ as a homogenous bulk material so one can expect only one value of SAW. The obtained results are presented in the Table 2.

**Table 2:** The values of the RW velocities calculated on base of available literature data of elastic tensor coefficient.

	LDA^15^	PBE-VDW^15^	DFT^25^	Experiment^14^
υ_SAW_ (m/s)	1,640	1,580	1,580	1,540

The calculated velocities are closer to velocity of RW_2_ mode and they are similar within the accuracy of about 6%. What is more interesting we found their dispersion relations as linear function of angle Θ (or wave vector *q*). This support additionally our previous statement that only Rayleigh waves can propagate on the bulk bismuth telluride surface.

**Figure 6:**
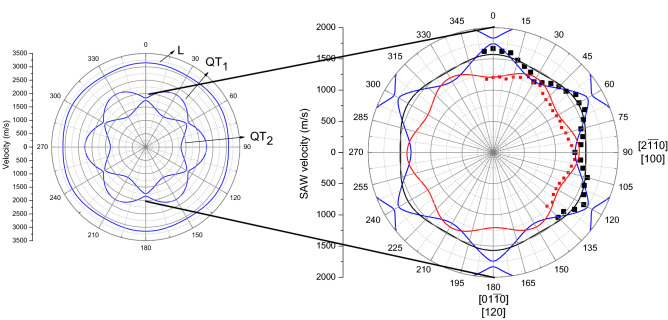
Elastic anisotropy of Bi_2_Te_3_ calculated from the experimental data in Ref.^14^.

The angular anisotropy of such SAW have been determined experimentally (points on Fig. 6) and based on FEM simulations (lines on Fig. 6). According to the FEM simulation the main directions of SAW propagation on (0001) plane had been determined. The structure of investigated material is trigonal so the typical triple symmetry on angular dependence on (0001) plane is observed (Fig. 6).

Numerous works have been carried out to clarify the influence of twinning on properties of topological insulators including stacking sequences, thickness and composition of layers in model structures, interface coherence, surface termination and morphology^26–30^.

The question is still open why two Rayleigh waves are observed from the single crystal of Bi_2_Te_3_.

**Figure 7:**
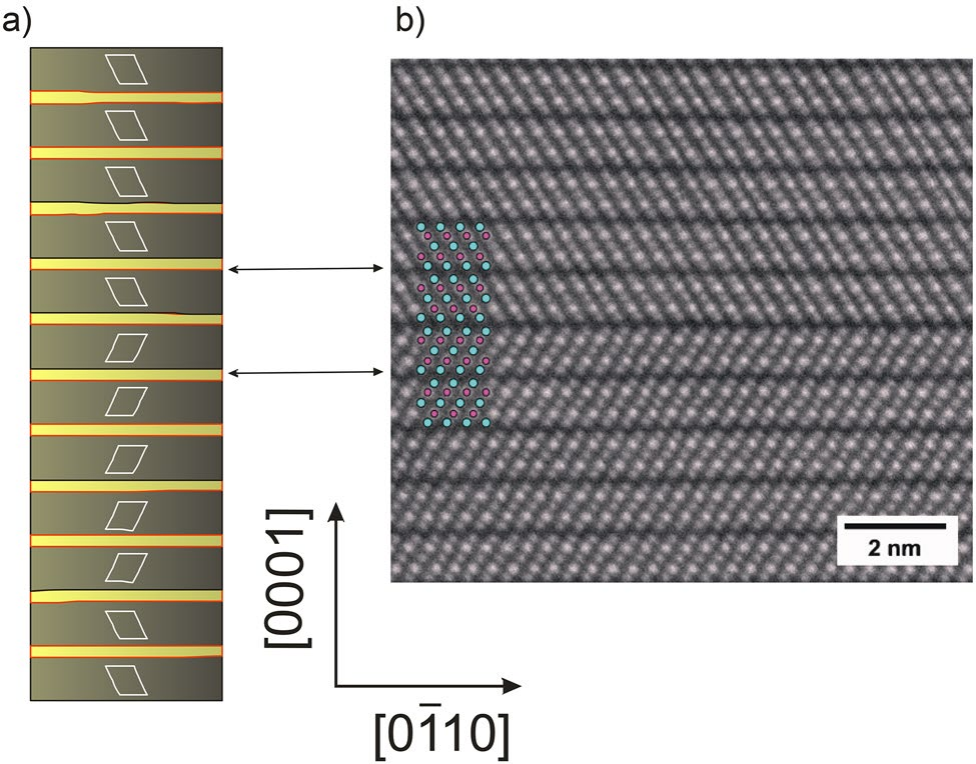
FEM unit cell with marked regions of different orientations of atoms (left and right oriented rhombs (**a**). HAADF-STEM image of basal twin of Bi_2_Te_3_. High intensity peaks correspond to Bi atomic columns; low intensity peaks correspond to Te atomic columns. Reproduced from Medlin et al.^28^, with the permission of AIP Publishing (**b**).

**Figure 8:**
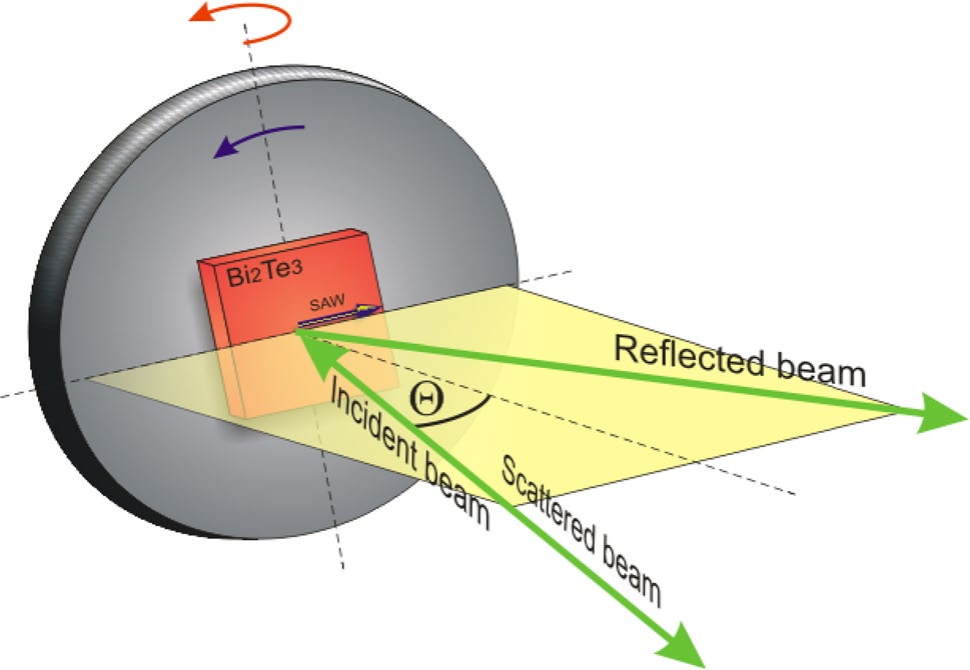
Schematic illustration of the sample holder for the surface phonon investigations.

To explain this we have to adopt results of more sensitive, than ours experimental and simulations methods. Medlin et al.^29,30^ investigated the structure of the (0001) basal twin boundary in Bi_2_Te_3_ with the use of high resolution electron diffraction (HAADF-STEM) supported by ab initio calculations. The interfacial atomic structure measurements showed that it is possible for the twin interface to be located at one of three distinct locations: at the Te(2) layer, the Bi layer, or the Te(1) layer. In the Fig. 8 the FEM unit cell with marked regions of different orientations of atoms relevant to the results of Medlin et al.^28^. The clear twin boundary can be seen in Fig. 7b so the incident light is passing through the two type of domains (marked as a left and right turn rhombs in the Fig. 7a. This results in observation of two separate surface acoustic waves in presented experiment.

In conclusions, we believe the present work significantly improves the comprehension of mechanical properties of single crystal topological insulator. For the first time Brillouin scattering experiment was used to study the surface elastic properties of Bi_2_Te_3_ single crystal. Two Rayleigh waves were observed. Both surface excitation show linear dispersion and thus they are Rayleigh waves. This distinguishes Bi_2_Te_3_ from other metals and, in our opinion, results from its unique crystalline structure.

FEM unit cell applied in simulation reflect very well observed in experiment dispersion and anisotropy of two Rayleigh waves. Replacement of “empty” VdW regions by an air layer is reliable in terms of the size of the oxygen and nitrogen molecules.

We believe our results reliably complement the existing data on elastic properties of Bi_2_Te_3_ single crystal. This is important when the transition from bulk elastic properties (3D) to thin films behavior is considered (2D). Then the change from linear to nonlinear phonon dispersion is observed and this in turn may be useful for future applications of topological insulators in spintronics or thermo electric energy conversion. We are aware that a complete analysis of elastic properties and phonons dynamic in 3D and 2D material requires new experiments. First of all it would be very informative to investigate the several heterostructures semiconductor/thin films of topological insulator with different thickness^31^. The quite challenging, but possible, would be experiments on non-supported thin film of Bi_2_Te_3_ or its exfoliated forms.

## Materials and methods

### Sample description

The Bi_2_Te_3_ crystals (space group $R\overline{3}m$, point group $\overline{3}m$) were synthesized using modified flux technique to attain large size of the samples (see supplementary (SI) for details). The elastic stiffness tensor of point group $\overline{3}m$ contains six independent elastic constants: c_11_ = c_22_, c_33_, c_44_ = c_55_, c_66_ = (c_11 _− c_12_), c_12_, c_13_ = c_23_, c_14_ =  − c_24_ = c_56_.

So far elastic properties of Bi_2_Te_3_ have been studied experimentally using the ultrasonic method^13,14^ and nanoindentation^15^ supported by density functional theory (DFT)^25^, local density approximation (LDA)^15^ and Perdew–Burke–Ernzerhof (PBE) algorithm^15^. In the Table 3 we have collected elastic constants obtained with different methods.

**Table 3:** Elastic constants of Bi_2_Te_3_ obtained with different methods.

	c_11_	c_33_	c_44_	c_12_	c_13_	c_14_
EXP^14^	74.4	47.7	27.4	21.7	27.0	13.3
LDA^15^	81.5	56.4	42.7	22.2	31.2	19.4
PBE^15^	78.3	35.7	35.5	13.8	23.2	20.7
DFT^25^	65.4	50.7	26.5	14.0	19.0	10.9

### Experimental setup

The propagation of surface acoustic waves on the (0001) plane of Bi_2_Te_3_ single crystal was studied using a six-pass, tandem Brillouin spectrometer (J. Sandercock system, Table Stable Ltd., Switzerland) which ensures a contrast of 10^15^^32^. The source of light was a Nd:YAG single-mode diode-pumped laser, emitting the second harmonics of light of the length *λ*_0_ = 532 nm with the power of 200 mW (Excelsior, Spectra Physics). Measurements were made in the backscattering geometry. The SAW energy is represented by the Brillouin frequency shift *Δf* of the inelastically scattered laser beam. The wave vector *q* of the investigated SAW varied from 0.0008 to 0.0233 nm^−1^. For the measurements of angular dependence of the frequency of SAWs on the investigated plane of the single crystal the Bi_2_Te_3_ single crystal was mounted on the rotation stage (see Fig. 8). Both incident and scattered light were polarized vertically. A detailed description of the experimental setup can be found in Refs.^33,34^.

Vertical rotation (red arrow) allowed for dispersion relation measurements. The velocity of surface acoustic waves *V*_SAW_ depends on angle Θ between scattered light and normal to sample plane as follows:

1 $$ \upsilon_{SAW} = \frac{{\Delta f_{SAW} \lambda_{0} }}{2\sin \Theta } $$

where Θ is the scattering angle, *Δf*_*SAW*_ is the Brillouin frequency shift observed in experiment and λ_0_ = 532 nm. The wave vector values are calculated from the expression:

2 $$ q = \frac{4\pi sin\Theta }{{\lambda_{0} }} $$

Rotation of the sample around the axis perpendicular to sample plane (blue arrow) for fixed Θ gives the information about anisotropy of surface acoustic waves (SAW) in plane under investigation.

### FEM

The calculations of the dispersion relation and angular dependence of frequency for the surface phonons propagating in the studied Bi_2_Te_3_ single crystal were performed using Finite Element Method (FEM), as implemented in the COMSOL Multiphysics software^35^. FEM is based on the Floquet–Bloch theory which provides a strategy to obtain a set of solutions of a linear ordinary equations^36^.

## Supplementary information

Supplementary Information.
