# Supplementary material for: Surface phonons in topological insulator Bi2Te3 investigated by Brillouin light scattering
Source: Sci Rep. 2020 Jul 16;10:11812. doi: 10.1038/s41598-020-68690-z (PMC7367295; doi:10.1038/s41598-020-68690-z)
Supplement: Supplementary file 1 — Supplementary Information. [file 41598_2020_68690_MOESM1_ESM.docx]

**Supporting Informations**

Surface phonons in topological insulator Bi_2_Te_3_ investigated by Brillouin light scattering

1. Trzaskowska^*^, B. Mroz

Faculty of Physics, Adam Mickiewicz University, Uniwersytetu Poznańskiego 2

61-614 Poznan, Poland

**SI 1 Crystal growth**

Flux zone growth technique was preferred over convention chemical vapor transport since flux zone growth is capable of producing perfect stoichiometric ratios (2:3) and prefer Bi_2_Te_3_ crystals from being contamined by halides which is commonly used in chemical vapor transport. The growth was performed at temperatures below 586^o^C under 10^-7^torr pressures. Cooling rates were selected to be slow to allow for good crystallization (~2C/hour). All the metal and chalcogen precursors were 6N purity and was in ingot (nugget) and was sealed in a quartz ampoule. The Bi_2_Te_3_ crystals were purchased from 2D Semiconductors Inc., Scottsdale, AZ USA [1,3].

**SI 2 Finite Element Method (FEM) simulations**

The dispersion relation and angular dependence of frequency for the surface phonons propagating in the studied Bi_2_Te_3_ single crystal was obtained by Finite Element Method (FEM) code of COMSOL Multiphysics software [4]. The investigated crystal is a bulk material so the elementary unit cell for simulation should be treated as homogenous block with well-defined elastic properties and density.

The Floquet-Bloch theory provides a strategy to obtain a set of solutions of a linear ordinary equations which is the key issue of finite element method (FEM) [5]. For the walls the Bloch-Floquet [6,7] periodic boundary conditions were specified for each of the three components of deformation:

$$u exp\left[ i\left( k_{x}x+k_{y}y+k_{z}z \right) \right]$$

$$v exp\left[ i\left( k_{x}x+k_{y}y+k_{z}z \right) \right]$$

$$w exp\left[ i\left( k_{x}x+k_{y}y+k_{z}z \right) \right]$$

where *u*, *v*, and *w* stand for the components of deformation in the rectangular system of coordinates, and the wave vector $\mathbf{k}=\left( k_{x}, k_{y},k_{z} \right)=(k_{x}cos\alpha, k_{y}cos\beta, k_{z}cos\gamma)$. In the above expressions, *α*, *β*, and *γ* are the angles made by the wave vector and the *x*, *y*, and *z* axes of the coordinate system. For the Rayleigh-type SAW, it is assumed that *k_z_* is zero (the wave vector of the surface wave is in the *xy* plane), while $cos\beta=sin\alpha$. The above conditions were applied to the walls perpendicular to the directions $[0\bar{1}10]$ and $[2\bar{1}\bar{1}0]$.

Bi_2_Te_3_ is a bulk material but it is characterized by layered structure [8,9]. According to this fact in the first approach for FEM calculations the unit cell used in simulations is a block of bismuth telluride (presented on Figure 6). Relevant literature provides information on the elastic constants of the bismuth telluride using different methods [10-13]. The elastic constants available in the literature are given in general form typical for a given symmetry and do not include the plane on which surface waves propagate. The investigated surface of the sample was (0001) so all components of elastic tensor coefficient have been transformed to the (0001) plane. First, it should be noted that the all direction on the trigonal plane (0001) should be transformed to the cubic notation. Next the elastic tensor $c_{ij}$ can be transformed according to the equation [14-16]:

$$c_{ij}^{'}=Rc_{ij}R^{T}$$

where:

$$R=\left[\begin{matrix} a_{11}^{2} & a_{12}^{2} & a_{13}^{2} & 2a_{12}a_{13} & 2a_{13}a_{11} & 2a_{11}a_{12} \\ a_{21}^{2} & a_{22}^{2} & a_{23}^{2} & 2a_{22}a_{23} & 2a_{23}a_{21} & 2a_{21}a_{22} \\ a_{31}^{2} & a_{32}^{2} & a_{33}^{2} & 2a_{32}a_{33} & 2a_{33}a_{31} & 2a_{31}a_{32} \\ a_{21}a_{31} & a_{22}a_{32} & a_{23}a_{33} & a_{22}a_{33}+a_{23}a_{32} & a_{21}a_{33}+a_{23}a_{31} & a_{22}a_{31}+a_{21}a_{32} \\ a_{31}a_{11} & a_{32}a_{12} & a_{33}a_{13} & a_{13}a_{32}+a_{12}a_{33} & a_{13}a_{31}+a_{11}a_{33} & a_{11}a_{32}+a_{12}a_{31} \\ a_{11}a_{21} & a_{12}a_{22} & a_{13}a_{23} & a_{12}a_{23}+a_{13}a_{22} & a_{13}a_{21}+a_{11}a_{23} & a_{11}a_{22}+a_{12}a_{21} \end{matrix}\right]$$

and *a* is direction cosine matrix:

$$a= \left[ \begin{matrix} a_{11} & a_{12} & a_{13} \\ a_{21} & a_{22} & a_{23} \\ a_{31} & a_{32} & a_{33} \end{matrix} \right]$$

For the bottom wall of unit cell the boundary conditions were fixed. The height of the elementary cell used in the simulations was correlated with the wavelength of the acoustic wave propagating in the sample. According to the Farnell theory [17] the surface acoustic Rayleigh wave λ_R_ penetrates material into the depths of about two its wavelength. Taking to account that the experimental wave vectors accessible in our experiment are ranging from 0.0008 to 0.0233 nm^-1^ (which corresponds the Θ changes from 2^0^ to 80^0^) the λ_R_ is 7600 nm and 270 nm, respectively. It is well known that for the edge values of Θ the quality of Brillouin spectra is decreasing, so we present the results obtained in the Θ ranging from 30^0^ to 70^0^ which result in λ_R_ varying between 520 and 290 nm. In our simulation we decide to set the height of unit cell to be 2000 nm. Such height ensured the absence of the unwanted reflections of simulated waves from the bottom of cell and was a compromise between height and computing capabilities of the workstation.

In order to find the velocity distribution of both L and T acoustic waves in (0001) plane the Christoffel equation was solved:

$$\left| C_{ijkl}l_{j}l_{k}-\delta_{il}\rho v^{2} \right|=0$$

$$det\left| \begin{matrix} \left( c_{11}a_{1}^{2}+c_{66}a_{2}^{2} \right)-\rho v^{2} & \frac{\left( c_{11}\cdot+c_{12} \right)}{2}a_{1}a_{2} & 2c_{14}a_{1}a_{2} \\ \frac{\left( c_{11}+c_{12} \right)}{2}a_{1}a_{2} & \left( c_{11}a_{2}^{2}+c_{66}a_{1}^{2} \right)-\rho v^{2} & 0 \\ 2c_{14}a_{1}a_{2} & 0 & \left( c_{44}\left( a_{1}^{2}+a_{2}^{2} \right) \right)-\rho v^{2} \end{matrix} \right|=0$$

Where $a_{1}=cos\alpha, a_{2}=sin\alpha and$α is an angle between the phonon direction and axis [100] on the investigated plane.

## FEM unit cell

In the Figure S1 the transformation from hexagon to cuboidal symmetry required in FEM simulations is presented. Such transformation, from Miller to Miller-Bravis indices, ensures preservation of elastic properties of simulated unit cell.

Figure S1. Relation between hexagonal and cuboidal symmetry required in FEM.

**SI 3 Surface and pseudo surface waves**

The matter needs to be resolved whether both observed in our experiment mode (R1 and R2) are pure RW or the faster one is pseudo surface acoustic wave PSAW [18-19]. In such a case the key classification criterion is not only the relationship between velocity of the slowest transverse mode and surface wave but also the angular range of where the velocity criterion holds. In the anisotropic materials the velocity of Rayleigh wave (RW) is always lower from the slowest transverse bulk wave [17, 20] regardless its character –whether the wave is pure transverse or quasi transverse. In our case when the plane (0001) is considered all transverse modes contain off diagonal components of elastic constants $c_{ij},$ so they are quasi transverse. The existing so far research results indicate for only specific directions of the anisotropic medium where the phase velocity of pseudo surface wave is higher than the Rayleigh Wave.

**References**

# [1] Capper, P. *Bulk Crystal Growth: Methods and Materials*. *Springer Handbook of Electronic and Photonic Materials.* (eds Kasap S., Capper P.) (Springer Handbooks. Springer, Cham, 2017).

[2] <https://www.2dsemiconductors.com/bismuth-telluride-bi2te3/#description>.

[3] Steiner, H. et al. [Structure and composition of bis­muth telluride topological insulators grown by molecular beam epitaxy](https://journals.iucr.org/j/issues/2014/06/00/rg5074/index.html). [*J. Appl. Cryst.*](https://www.researchgate.net/journal/1600-5767_Journal_of_Applied_Crystallography) **47,** 1889-1900 (2014).

[4] COMSOL Multiphysics finite element software, COMSOLAB, Sweden.

[5] Gómez García, P. & Fernández-Álvarez, J.-P. Floquet-Bloch Theory and Its Application to the Dispersion Curves of Nonperiodic Layered Systems. *Mathematical Problems in Engineering* **2015,** 475364 (2015).

[6] Laude, V, *Phononic Crystals Artificial Crystals for Sonic, Acoustic, and Elastic Waves*, in Studies in Mathematical Physics **(**ed. [De Gruyter](https://www.degruyter.com/view/serial/129734) **26**, 2015).

[7] Trzaskowska, A., [Mielcarek,](https://www.scopus.com/authid/detail.uri?authorId=6603712369&amp;eid=2-s2.0-84885446112) S. & [Sarkar, J. Band gap in hypersonic surface phononic lattice of nickel pillars**.**](https://www.scopus.com/authid/detail.uri?authorId=14069003700&amp;eid=2-s2.0-84885446112) [*J. Appl. Phys.*](file:///C:\Users\Jarek\AppData\Local\Microsoft\Windows\E:\J.%20Appl.%20Phys) **114,** 134304 (2013).

[8] Zhang, H. et al. Topological insulators in Bi_2_Se_3_, Bi_2_Te_3_ and Sb_2_Te_3_ with a single Dirac cone on the surface. *Nature Physics* **5,** 438-442 (2009).

[9] Chen, Zhi-Gang**,** [Han, G. F.**,** Yang, L.**,** Cheng, L. & Zou, J. [Nanostructured thermoelectric materials: Current research and future challenge](https://www.sciencedirect.com/science/article/pii/S1002007112001384)**.**](https://www.sciencedirect.com/science/article/pii/S1002007112001384#!) [*Progress in Natural Science: Materials International*](https://www.sciencedirect.com/science/journal/10020071) **22**, 535-549 **(**2012).

[10] Akgös, Y. C., Saunders, G. A. & Sümengen, Z. Elastic wave propagation in Bi_1⋅60_Sb_0⋅40_Te_3_ and Bi_2_Te_3_. *J. Mater. Sci.* **7,** 279 -288 (1972).

[11] Jenkins, J. O., Rayne, J. A. & Ure, R. W. Jr. Elastic Moduli and Phonon Properties of Bi_2_Te_3_. *Phys. Rev. B* **5,** 3171 (1972), Erratum *Phys. Rev. B* **6,** 1609 (1972).

[12] Huang, B.-L. & Kaviany, M. *Ab initio* and molecular dynamics predictions for electron and phonon transport in bismuth telluride. *Phys. Rev. B* **77,** 125209 (2008).

[13] [Lamuta](https://www.sciencedirect.com/science/article/pii/S1359646216301646#!), C. et al. Mechanical properties of Bi_2_Te_3_ topological insulator investigated by density functional theory and nanoindentation. [*Scripta Materialia*](https://www.sciencedirect.com/science/journal/13596462) [**121**](https://www.sciencedirect.com/science/journal/13596462/121/supp/C)**,** 50-55 (2016).

[14] Newnham, R. E. *Properties of Materials: Anisotropy, Symmetry, Structure* (Kindle Edition). (Oxford University Press Inc., Oxford and New York, 2005).

[15] Espinoza-Beltrán, F. J. et al. Simulation of vibrational resonances of stiff AFM cantilevers by finite element methods. *New Journal of Physics* **11,** 083034 (2009).

[16] Lieberman, D. S. & Zirinsky, S. [A simplified calculation for the elastic constants of arbitrarily oriented single crystals](http://journals.iucr.org/q/issues/1957/03/00/a01970/a01970.pdf)**.** Acta Crystallogr. **9,** 431 **(**1956).

[17] Farnell, G. W. *Properties of elastic surface waves* in Physical Acoustics 6, 109-166 (Academic Press, New York, 1970).

[18] Kundu, N. *Ultrasonic Nondestructive Evaluation Engineering and Biological Material Characterization*. (CRC Press, 2004).

[19] Mutti, P. *Advances of Acoustic Microscopy.* (ed. Briggs, A.) 249-300 (Springer, 1995).

# [20] Auld, B. A. *Acoustic fields and waves in solids.* (Willey, 1973).
